# A bibliometric and visualization analysis of global research on postherpetic neuralgia from 2000 to 2022: A review

**DOI:** 10.1097/MD.0000000000034502

**Published:** 2023-11-10

**Authors:** Yujun He, Jiujie He, Furui Miao, Yushan Fan, Fangzhi Zhang, Zibin Wang, Yu Wu, Yiping Zhao, Pu Yang

**Affiliations:** a Faculty of Acupuncture, Moxibustion and Tui Na, Guangxi University of Chinese Medicine, Nanning city, People’s Republic of China; b Graduate School, Guangxi University of Chinese Medicine, Nanning city, People’s Republic of China.

**Keywords:** bibliometric analysis, CiteSpace, global trend, postherpetic neuralgia

## Abstract

Postherpetic neuralgia (PHN) represents a notable clinical challenge as it is the most prevalent and severe complication of herpes zoster (HZ). The primary objective was to investigate the current research status and hotspots of PHN research during the period from 2000 to 2022. The literature pertaining to PHN was gathered through the utilization of the Web of Science Core Collection, spanning from January 2000 to December 2022. The software, CiteSpace version 6.2.R2, was employed to produce visual depictions of publications related to PHN across various dimensions such as year, country/region, institution, journal, author, keyword, and reference. This study involved a total of 3505 papers. The USA held a dominant position in the production of scholarly articles. Argentina exhibited the highest frequency of participation in international collaboration. Out of all the institutions, Pfizer exhibited the highest degree of productivity. Harvard University exhibited the highest frequency of participation in international collaboration. The *Pain* exhibited the most noteworthy productivity rate and citation count among all other journals. Ralf Baron was identified as the most productive author, whereas DWORKIN RH attained the highest citation count. Contemporary scholarly investigations are predominantly centered on identifying risk factors, devising preventative measures, and exploring novel and secure methods of pain management. The current investigation has revealed the focal areas and patterns of studies pertaining to PHN. Presently, the research in this field is focused on identifying the risk factors and preventive measures for PHN, alongside exploring novel and secure pain management strategies.

## 1. Introduction

Postherpetic neuralgia (PHN) is a syndrome described as zoster-associated pain persisting for more than 3 months after resolution of an initial herpes zoster (HZ) rash (“shingles”).^[1]^ This condition is considered to be the most prevalent and challenging complication associated with HZ infection.^[2]^ According to reports, around 75% of senior patients who contract HZ are likely to experience it.^[3]^ The incidence of it was observed to be 38.1%, 27.0%, and 19.0% at 1 month, 3 months, and 6 months, respectively, following the onset of zoster. It is noteworthy that the incidence of PHN seems to be on the rise.^[4]^ Advanced age, the manifestation of a prodrome, severe rash, and severe pain are established risk factors for the development of PHN.^[5,6]^ The intensity of PHN exhibit a positive correlation with advancing age, and are linked to the progressive decline of cell-mediated immunity against varicella zoster virus.^[7]^ The mechanisms underlying PHN remain incompletely understood, however, insights have been gleaned from both animal and clinical investigations. PHN can be further classified into 2 distinct subcategories, namely the irritable nociceptor model and the deafferentation model.^[8,9]^ There are numerous treatment modalities available for the management of PHN, encompassing pharmacological and interventional therapies.^[10,11]^ It is not uncommon for a combination of these therapeutic approaches to be employed in the management of PHN.^[12]^ Nevertheless, extant treatment options are not without their limitations.^[9]^ It significantly affects the occupational and daily functioning of patients, while also placing a considerable economic burden on families and society. Additional investigation is necessary to comprehensively examine this ailment.^[13]^

Bibliometric analysis is a quantitative method that involves the examination of diverse publications related to a particular subject in order to assess the research status using mathematical and statistical techniques.^[14,15]^ In contrast to traditional research methods such as meta-analyses, study reviews, and experimental or clinical research, this approach confers advantages by enabling a more thorough understanding of key research findings among research groups.^[16–18]^ The present analysis utilizes citation frequency as a metric, which refers to the frequency at which a scholarly publication is referenced by other researchers.^[10]^ Bibliometric analysis is a widely recognized method for assessing the attributes and academic influence of a particular field of study. This approach is widely employed to evaluate the merit of a given discipline and offer valuable insights into the advancement and development of a field.^[19,20]^

In recent years, this particular methodology has been employed within the primary care and public health domains to investigate a range of ailments associated with pain, such as dysmenorrhea,^[21]^ sarcopenia associated with osteoporosis,^[22]^ shoulder pain,^[23]^ cancer pain,^[24]^ and myofascial pain syndrome.^[25]^ Several academic assessments have discussed the clinical administration, pathophysiological mechanisms, and epidemiological features of PHN.^[26]^ Furthermore, a bibliometric examination of HZ revealed that PHN is presently the primary area of research interest, warranting additional investigation.^[27]^ This study utilized CiteSpace to conduct a visualization analysis on interrelated references within PHN sourced from the Web of Science (WOS) database. The main aims of the study were to investigate areas of high research activity and trends in development, and to provide a benchmark for future research endeavors. CiteSpace possesses the capacity to produce knowledge maps via bibliometric examination and visualization by creating nodes and links.

## 2. Methods

### 2.1. Data sources and search strategy

**Figure 1.**
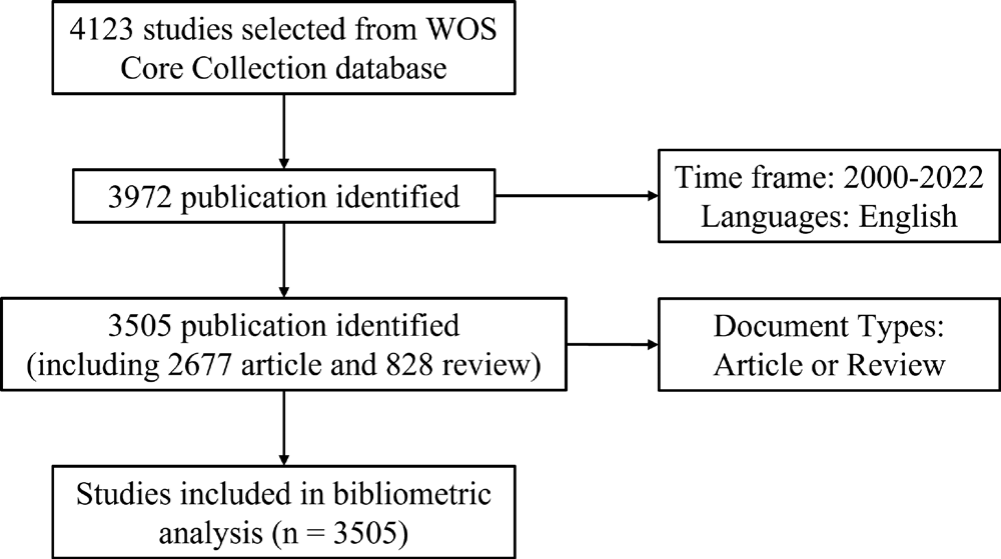
A study flow diagram of the literature screening and selection processes from WoS Core Collection. WOS = Web of Science.

The WOS Core Collection was chosen as the primary data source for acquiring comprehensive and authoritative articles. The research duration spanned from January 1, 2000 to December 31, 2022. We have consulted analogous scholarly works and restricted the range of document genres to solely encompass articles and reviews.^[28,29]^ The search criteria were defined as TS = (PHN) or (neuralgia, postherpetic) or (post-herpetic neuralgia) AND DT = (Article OR Review). In total, 3505 papers were retrieved, consisting of 2677 articles and 828 reviews. Figure 1 shows the flowchart of literature screening. The textual data pertaining to the records and references of each of these papers was exported in the form of TXT files.

The papers were gathered and analyzed independently by 2 authors (PY and JH). Any questions about the eligibility of the papers will be forwarded to a third reviewer (FZ). CiteSpace was utilized to conduct the analysis. The network visualization results were analyzed to investigate the leading institutions, authors, journals, countries/regions, keywords, and references. The following settings were made for CiteSpace: method (LLR), time slicing (January 2003—April 2022), years per slice (1), term source (all selection), node type (choose one at a time), selection criteria (top 50 objects), and pruning (pathfinder).

## 3. Results

### 3.1. Analysis of annual publications

**Figure 2.**
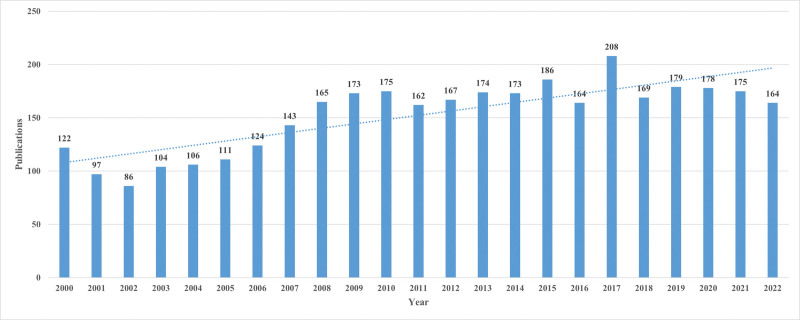
Annual publications from 2000 to 2022 and the time trend of PHN. PHN = postherpetic neuralgia.

Figure 2 was generated using Excel software after importing data from the WOS Core Collection. Figure 2 revealed that prior to 2007, the quantity of publications was below 150. However, subsequent to 2008, the number of publications has surpassed 150. The year 2002 recorded the minimum number of publications, which was 86, while the maximum number of publications was observed in 2017, which was 208. The figure illustrated that PHN has maintained a consistent level of significance over a period of 23 years and exhibits an upward trend.

### 3.2. Analysis of productive countries/regions and institutions

**Figure 3.**
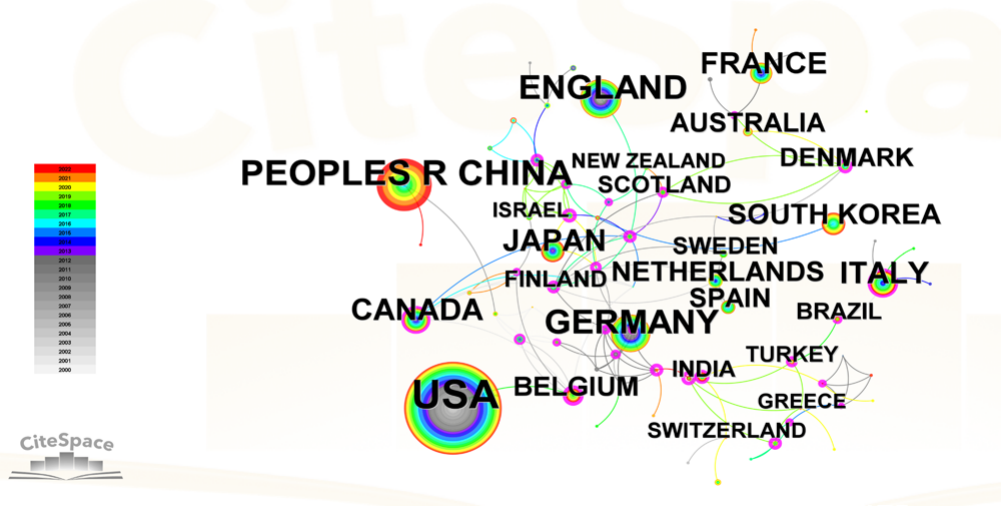
Citespace network map of countries/regions. Each node represents a country or region, and its magnitude indicates the number of publications from that country/region. The thickness of the lines indicates the intensity of the relationship between the regions/countries, which are represented by the connections between the nodes. The gray ring in the inner circle represents publications from the earliest year, while the red ring in the outer circle represents publications from the most recent year.

Publications pertaining to PHN were authored by 58 distinct countries/regions. The USA was responsible for the majority of publications, comprising a total of 1323 publications. Table 1 showed that China secured the second position with 461 publications, while the UK, Germany, and Japan followed with 358, 335, and 201 publications, respectively. The representation of international collaboration among countries/regions was illustrated in Figure 3. The conspicuous lack of global cooperation is a probable factor contributing to the relatively low centrality ratings of most countries/regions. Argentina demonstrated the most noteworthy degree of cooperation, as evidenced by its centrality score of 0.70. Panama and Philippines followed closely behind with scores of 0.67 and 0.60, respectively. The Czech republic and Norway also exhibited a certain level of cooperation, with centrality scores of 0.59 and 0.42, respectively.

**Table 1:** Top 10 countries/regions for publications of PHN.

Rank	Countries/Regions	Publications	Centrality
1	USA	1323	0.04
2	PEOPLES R CHINA	461	0.04
3	ENGLAND	358	0
4	GERMANY	335	0
5	JAPAN	201	0
6	CANADA	198	0.10
7	ITALY	190	0.13
8	FRANCE	164	0.09
9	SOUTH KOREA	123	0
10	NETHERLANDS	106	0.02

PHN = postherpetic neuralgia.

**Figure 4.**
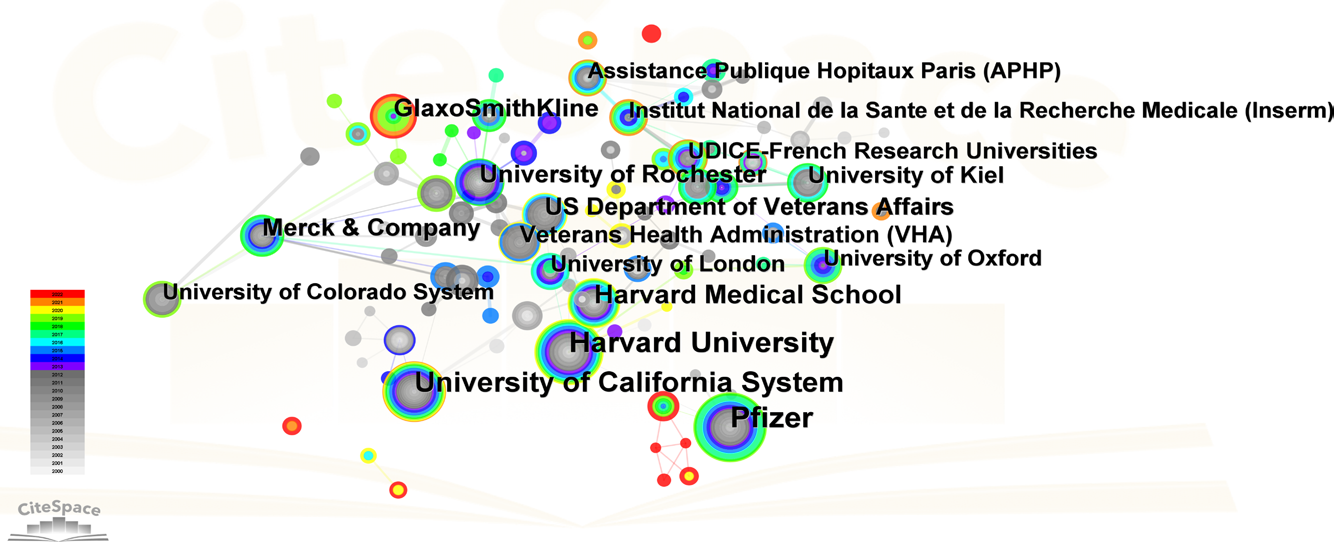
Institutional network map from Citespace. Each node represents an institution, and its magnitude indicates the number of publications that institution has produced. The thickness of the lines indicates the intensity of the relationship between the regions/countries, which are represented by the connections between the nodes. The gray ring in the inner circle represents publications from the earliest year, while the red ring in the outer circle represents publications from the most recent year.

The process of distributing academic articles related to PHN was facilitated by the involvement of 117 academic institutions. The network of institutional collaboration was depicted in Figure 4. Out of the top 10 institutions, 8 were situated in the USA, 1 in the UK, and 1 in Germany. Based on the information provided in Table 2, Pfizer emerged as the leading entity with 151 publications, trailed by Harvard University with 141 publications, University of California System with 128 publications, Harvard Medical School with 83 publications, and University of Rochester with 67 publications. In terms of centrality, the highest rank was obtained by Harvard University (0.47), followed by Merck & Company (0.44), Boston Children Hospital (0.34), Grunenthal Group (0.23), and University of Rochester (0.22) as presented in Figure 4.

**Table 2:** Top 10 institutions for publications on PHN.

Rank	Institutions	Countries/Regions	Publications	Centrality
1	Pfizer	USA	151	0.18
2	Harvard University	USA	141	0.59
3	University of California System	USA	128	0.15
4	Harvard Medical School	USA	83	0.03
5	University of Rochester	USA	67	0.22
6	GlaxoSmithKline	UK	65	0
7	Merck & Company	USA	57	0.44
8	US Department of Veterans Affairs	USA	57	0.15
9	University of Kiel	Germany	50	0
10	Veterans Health Administration (VHA)	USA	49	0

PHN = postherpetic neuralgia.

### 3.3. Analysis of journals and co-cited journals

The findings of the study on PHN were published in a collective of 906 scholarly journals. According to Table 3, the *Pain* has the highest number of publications, totaling 167. This is followed by the *Clinical Journal of Pain* with 95 publications, the *Pain Medicine* with 90 publications, the *Pain Physician* with 70 publications, and the *Journal of Pain Research* with 65 publications. As per the JCR 2021 criteria, it was observed that 3 journals from the top ten were assigned to the first quartile (Q1), whereas 4 journals were placed in the second quartile (Q2). Three journals were classified in the third quartile (Q3), while none of them were allocated to the fourth quartile (Q4).

**Table 3:** Top 10 journals for publications on PHN.

Rank	Frequency	Journal	IF (2021)	JCR	H-index	Country
1	167	Pain	7.926	Q1	258	USA
2	95	Clinical Journal of Pain	3.423	Q2	126	USA
3	90	Pain Medicine	3.637	Q2	97	UK
4	70	Pain Physician	4.396	Q2	99	USA
5	65	Journal of Pain Research	2.832	Q3	49	UK
6	62	Journal of Pain	5.383	Q1	127	USA
7	53	Cochrane Database of Systematic Reviews	12.008	Q1	273	USA
8	51	Pain Practice	3.079	Q3	58	UK
9	167	European Journal of Pain	3.651	Q2	109	USA
10	95	Vaccine	4.169	Q3	184	Netherlands

PHN = postherpetic neuralgia.

**Figure 5.**
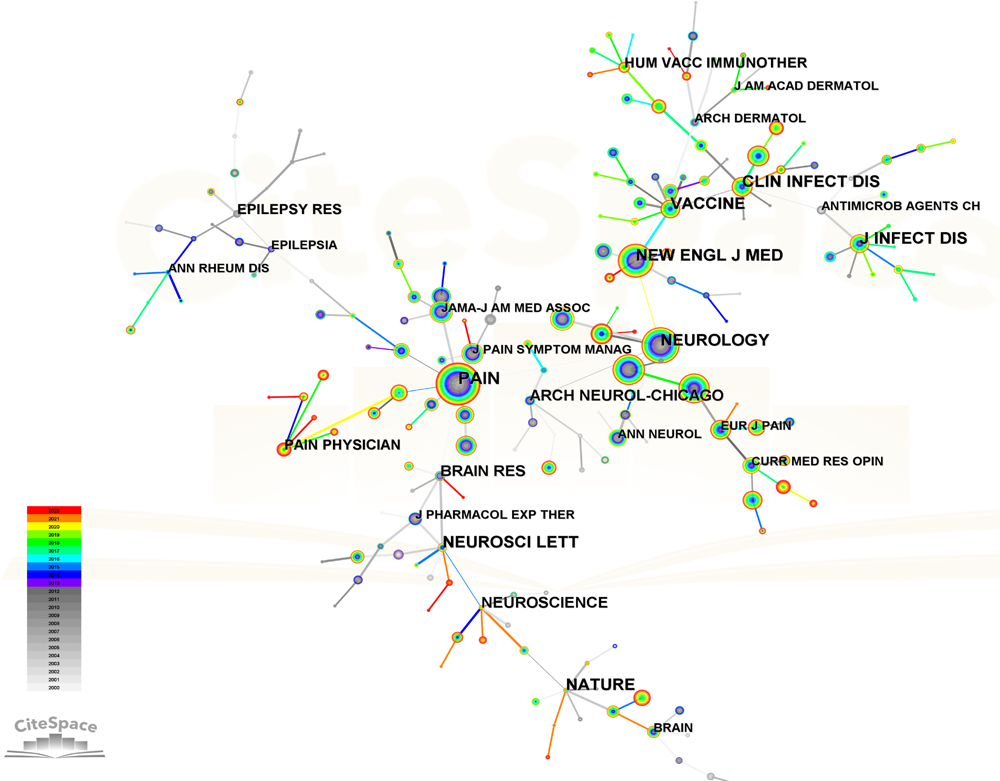
Citespace’s network map of co-cited journals. Each node represents a journal and the size of the node presents the number of publications of that journal. The thickness of the lines indicates the intensity of the relationship between the regions/countries, which are represented by the connections between the nodes. The gray ring in the inner circle represents publications from the earliest year, while the red ring in the outer circle represents publications from the most recent year.

The inquiry regarding PHN involved a total of 205 cited journals. The objective of performing a co-citation analysis on academic journals is to determine the most influential journals in a given field. The *Pain* prominent standing in the field was supported by its substantial citation count of 2508, which surpassed that of other notable publications such as the *Neurology* (1990 citations), the *New England Journal of Medicine* (1733 citations), the *Clinical Journal of Pain* (1423 citations), and the *Journal of Pain* (1252 citations). Following the JCR 2021 criteria, 8 out of the ten highest-ranking journals were categorized as Q1, while 2 of them were classified as Q2. It is noteworthy that none of the journals ranked within the top ten have been designated with a Q3 or Q4 classification. The preeminent journals in this field of study were identified as those that ranked within the top 10 periodicals and received a high number of citations, as demonstrated in Table 4 and Figure 5.

**Table 4:** Top 10 cited journals for publications on PHN.

Rank	Frequency	Centrality	Journal	IF (2021)	JCR	H-index	Country
1	2508	1.15	Pain	7.926	Q1	258	USA
2	1990	1.1	Neurology	11.800	Q1	364	USA
3	1733	0.85	New England Journal of Medicine	176.079	Q1	1030	USA
4	1423	0.21	Clinical Journal of Pain	3.423	Q2	126	USA
5	1252	0.17	Journal of Pain	5.383	Q1	127	USA
6	1118	0.11	JAMA-Journal of the American Medical Association	157.335	Q1	680	USA
7	1045	0	Lancet	202.731	Q1	762	UK
8	974	0.07	Journal of Pain and Symptom Management	5.576	Q1	140	Netherlands
9	848	0.16	European Journal of Pain	3.651	Q2	109	USA
10	840	0.5	Clinical Infectious Diseases	20.999	Q1	336	UK

PHN = postherpetic neuralgia.

### 3.4. Analysis of authors and co-cited authors

**Figure 6.**
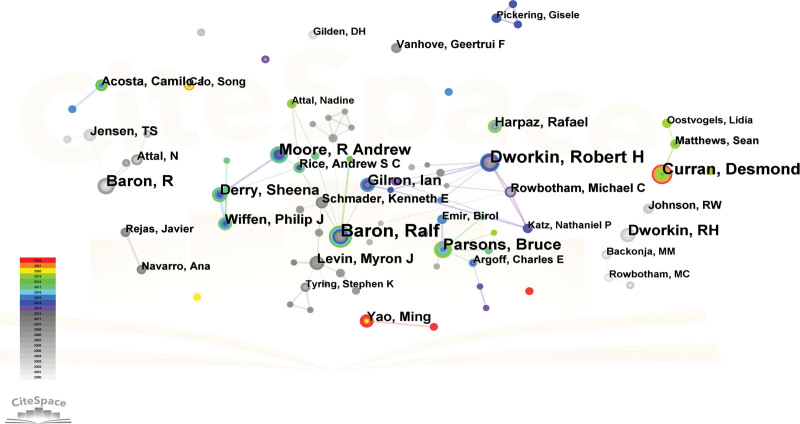
Authors’ network map from Citespace. Each node represents an author and the size of the node presents the number of publications of that author. The thickness of the lines indicates the intensity of the relationship between the regions/countries, which are represented by the connections between the nodes. The gray ring in the inner circle represents publications from the earliest year, while the red ring in the outer circle represents publications from the most recent year.

A total of 169 authors have made contributions to this specific field. As per the data presented in Table 5, Baron, Ralf affiliated with the University Medical Centre Schleswig-Holstein in Germany, has the highest count of published articles, amounting to 39. Following Baron, Ralf were Dworkin, Robert H with 32 articles, Curran, Desmond with 25 articles, Parsons, Bruce with 22 articles, and Moore, R Andrew with 22 articles. Figure 6 illustrated the authors’ collaboration. Price law is a commonly employed concept that elucidates the numerical correlation between the quantity of scientific literature and the number of scientists, as well as the relationship between scientists of varying levels of proficiency. The certification formula for the core author, as per Price law, can be expressed as M ≈ 0.749 √ Nmax.^[30]^ According to the formula, Ralf Baron has the highest number of posts among the authors, while M represents the minimum number of posts made by the core author. The value of M in this document is 4.68. Hence, it is possible to recognize authors as core authors if they have published a substantial quantity of articles rated with a 5. Based on the statistical findings, it was determined that a collective of 41 academics produced in excess of 5 scholarly articles each. Furthermore, these 41 principal authors were responsible for the publication of a cumulative total of 465 papers. The PHN field lacks a relatively stable core author group, as evidenced by the fact that the number of articles published by such authors constitutes <50% of the total.

**Table 5:** Top 10 authors for publications on PHN.

Rank	Frequency	Author	Country	Institution	Centrality
1	39	Baron, Ralf	Germany	Univ Klinikum Schleswig Holstein	0.12
2	32	Dworkin, Robert H	USA	Univ Rochester	0.08
3	25	Curran, Desmond	Belgium	GSK	0
4	22	Parsons, Bruce	USA	Beth Israel Deaconess Med Ctr	0.04
5	22	Moore, R Andrew	UK	Univ Oxford	0
6	21	Baron, R	Germany	Univ Kiel	0
7	18	Dworkin, RH	USA	Univ Rochester	0
8	17	Gilron, Ian	Canada	Queens Univ	0.09
9	17	Derry, Sheena	UK	Univ Oxford	0.02
9	14	Wiffen, Philip J	UK	UK Cochrane Ctr	0.01

PHN = postherpetic neuralgia.

**Figure 7.**
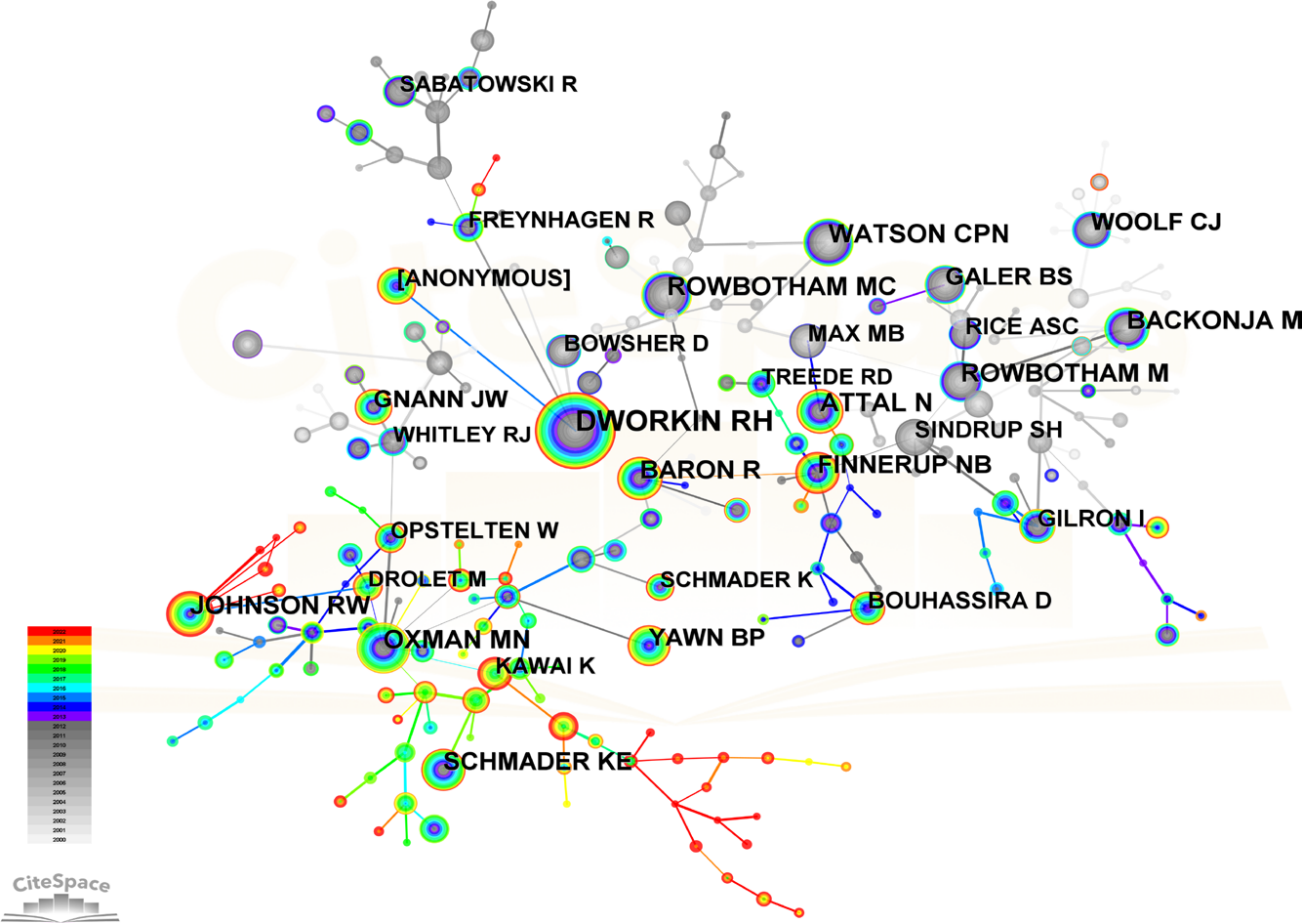
Citespace network map of co-cited authors. Each node represents an author and the size of the node presents the number of citations of that author. The thickness of the lines indicates the intensity of the relationship between the regions/countries, which are represented by the connections between the nodes. The gray ring in the inner circle represents publications from the earliest year, while the red ring in the outer circle represents publications from the most recent year.

The concept of co-cited authors pertains to a cluster of 2 or more authors who are cited jointly within a solitary publication, thus creating a co-citation association. The number of cited authors amounted to 230. DWORKIN RH, a scholar associated with the University of Rochester, has garnered the most citations in papers related to PHN, with a cumulative count of 1342 citations. DWORKIN RH was followed by ROWBOTHAM MC, OXMAN MN, WATSON CPN, and ATTAL N, who respectively received 618, 616, 601, and 576 citations. Table 6 and Figure 7 depicted a discernible co-citation network among numerous scholars.

**Table 6:** Top 10 cited authors for publications on PHN.

Rank	Frequency	Cited author	Country	Institution	Centrality
1	1342	DWORKIN RH	USA	Univ Rochester	0.49
2	618	ROWBOTHAM MC	USA	Univ Calif San Francisco	0.19
3	616	OXMAN MN	USA	VA Med Ctr	1
4	601	WATSON CPN	Canada	Univ Toronto	0.13
5	576	ATTAL N	France	Hop Ambroise Pare	0.06
6	505	JOHNSON RW	UK	Bristol Royal Infirm	0.09
7	504	BARON R	Germany	Univ Kiel	0.75
8	502	BACKONJA M	Spain	Hosp Gen Cataluna	0.22
9	490	ROWBOTHAM M	USA	Univ Calif San Francisco	0.49
10	474	FINNERUP NB	Denmark	Aarhus Univ	0.71

PHN = postherpetic neuralgia.

### 3.5. Analysis of keywords

**Figure 8.**
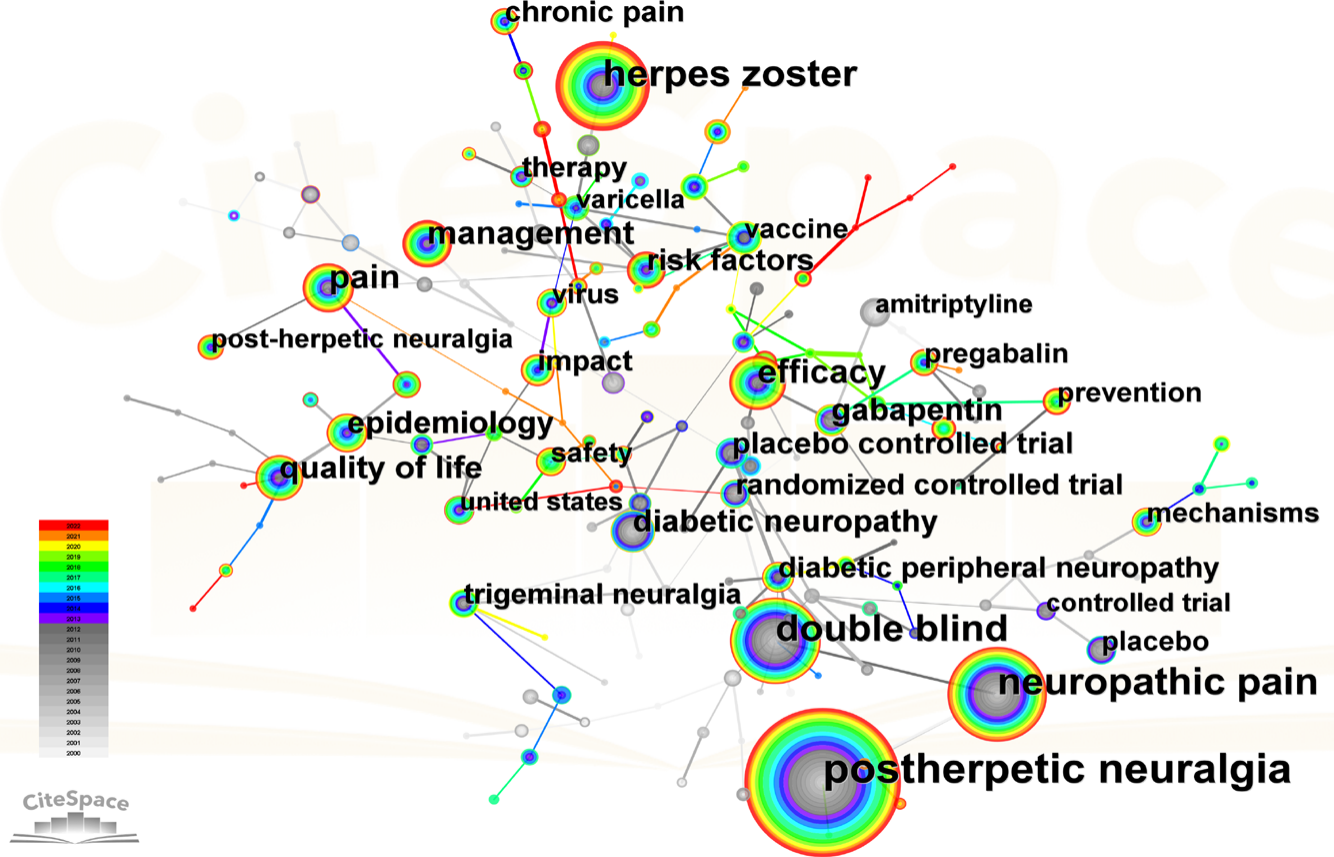
A keyword network visualization map. Each node represents a keyword and the size of the node presents the number of that keyword. The thickness of the lines indicates the intensity of the relationship PHN between the regions/countries, which are represented by the connections between the nodes. The gray ring in the inner circle represents publications from the earliest year, while the red ring in the outer circle represents publications from the most recent year.

**Figure 9.**
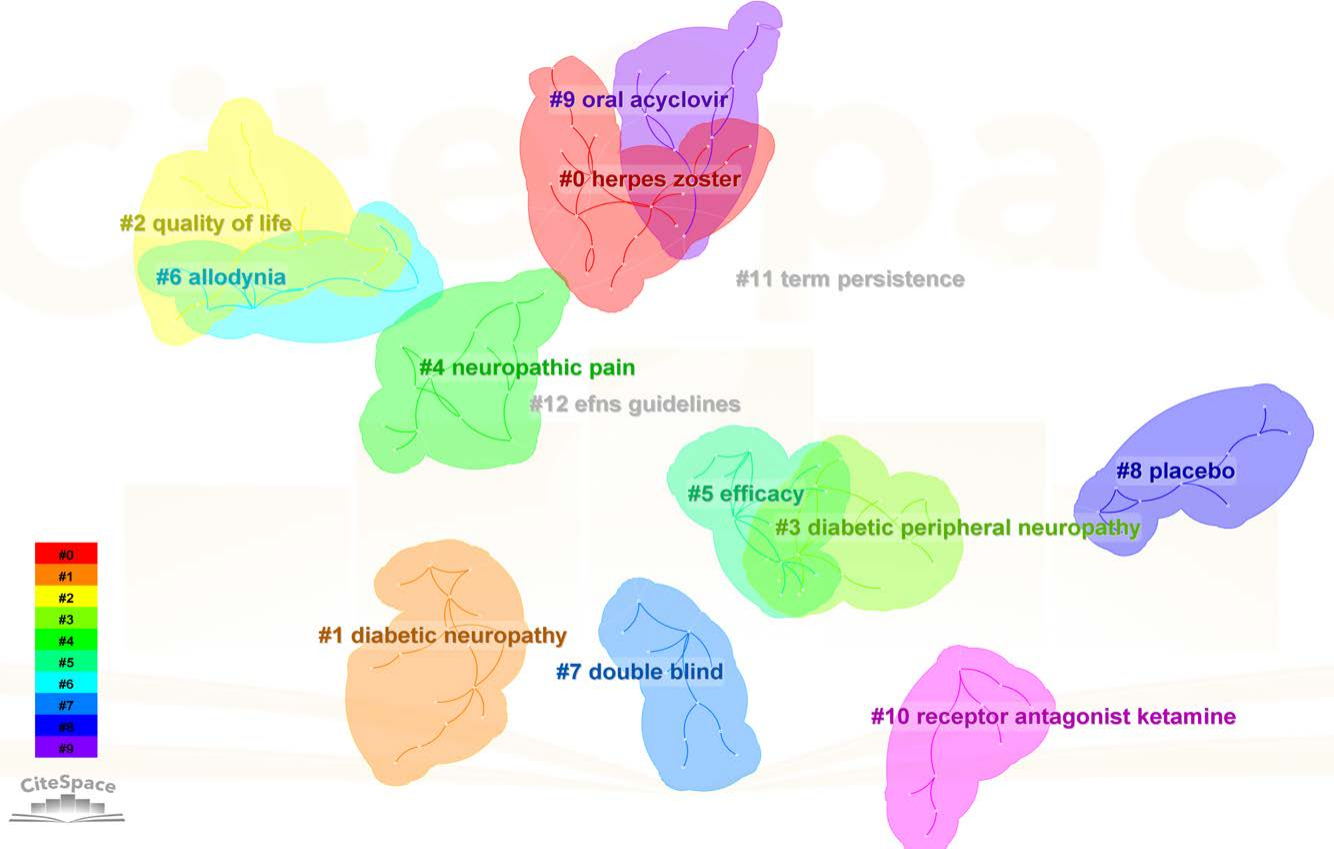
A cluster of keywords.

**Figure 10.**
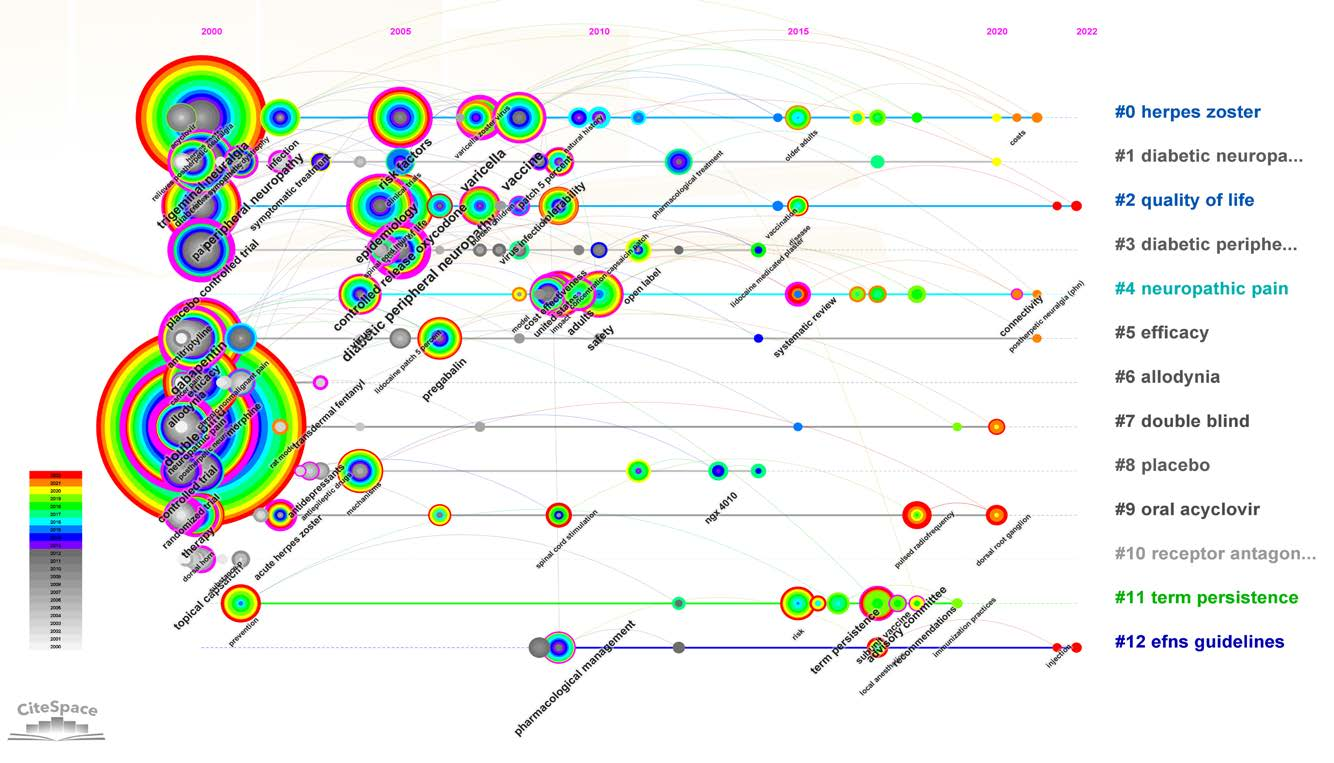
Timeline view of the keywords about PHN. PHN = postherpetic neuralgia.

**Figure 11.**
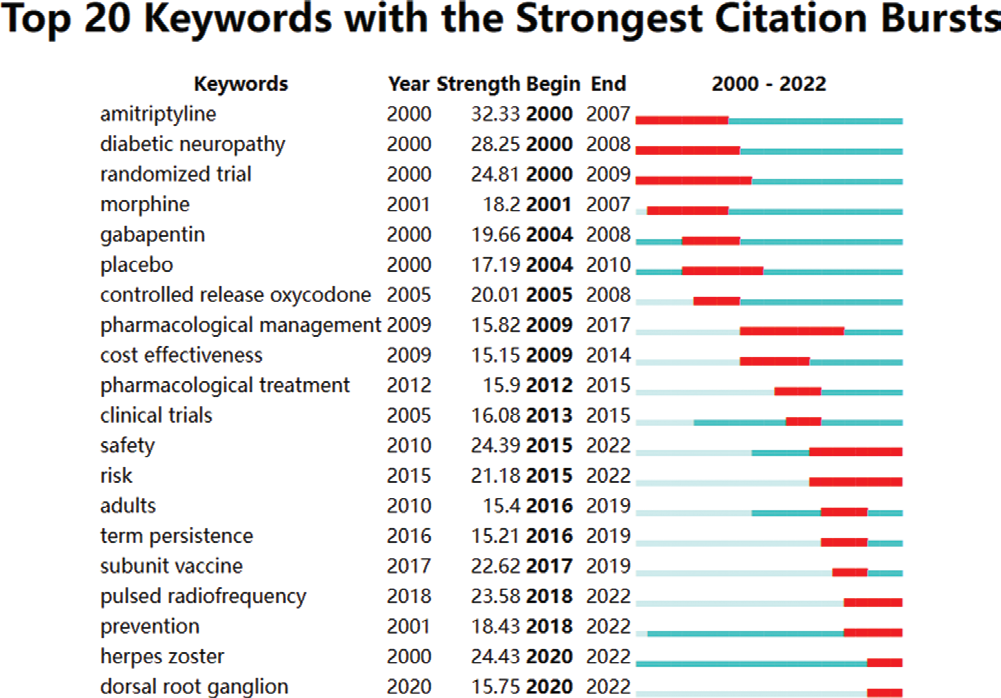
The top 20 keywords with the strongest citation bursts about PHN. PHN = postherpetic neuralgia.

The result of conducting a keyword analysis facilitates the identification of research centers and the prediction of emerging trends in a particular field. A total of 166 keywords were utilized in this particular field. Table 7 revealed that the most commonly utilized keywords were PHN (2503), neuropathic pain (1132), HZ (1015), double blind (960), and efficacy (457). The network map (Fig. 8) revealed that the keywords under consideration can be categorized into 12 distinct clusters: #0 HZ, #1 diabetic neuropathy, #2 quality of life, #3 diabetic peripheral neuropathy, #4 neuropathic pain, #5 efficacy, #6 allodynia, #7 double blind, #8 placebo, #9 oral acyclovir, #10 receptor antagonist ketamine, #11 term persistence, #12 efns guidelines (Fig. 9). Here a timeline showing the evolution of keywords over time (Fig. 10); the cluster words on the horizontal timeline view were on the right of Figure 10, and the left of Figure 10 was the evolution of related keywords from 2000 to 2022. Burst keywords are characterized by their frequent appearance within a compressed time frame. Through the process of reflecting upon recent research trends, there exists the potential to provide valuable insights into the evolution of research hotspots over time, as well as current and potential future research trends. The term “Strength” pertains to the degree of intensity of the burst, whereas “Begin” denotes the year when the burst initiated, and “End” signifies the year when it concluded. Figure 11 displayed the latest burst keywords, which included “pulsed radiofrequency,” “prevention,” “herpes zoster,” “dorsal root ganglion.” The “safety” and “risk” have been hotspots in this field since 2015.

**Table 7:** Top 20 keywords for publications on PHN.

Rank	Frequency	Keyword	Centrality
1	2503	Postherpetic neuralgia	0.02
2	1132	Neuropathic pain	0.09
3	1015	Herpes zoster	0.02
4	960	Double blind	0.7
5	457	Efficacy	0.25
6	410	Pain	0.17
7	398	Management	0
8	335	Quality of life	0.07
9	279	Gabapentin	0.4
10	279	Diabetic neuropathy	0.21
11	274	Epidemiology	0.29
12	234	Placebo controlled trial	0.68
13	221	Risk factors	0.2
14	203	Randomized controlled trial	0.86
15	200	Diabetic peripheral neuropathy	0.64
16	182	Chronic pain	0
17	163	Trigeminal neuralgia	0.13
18	157	Impact	0.11
19	152	Therapy	0.31
20	147	Safety	0.17

PHN = postherpetic neuralgia

### 3.6. Analysis of references

**Figure 12.**
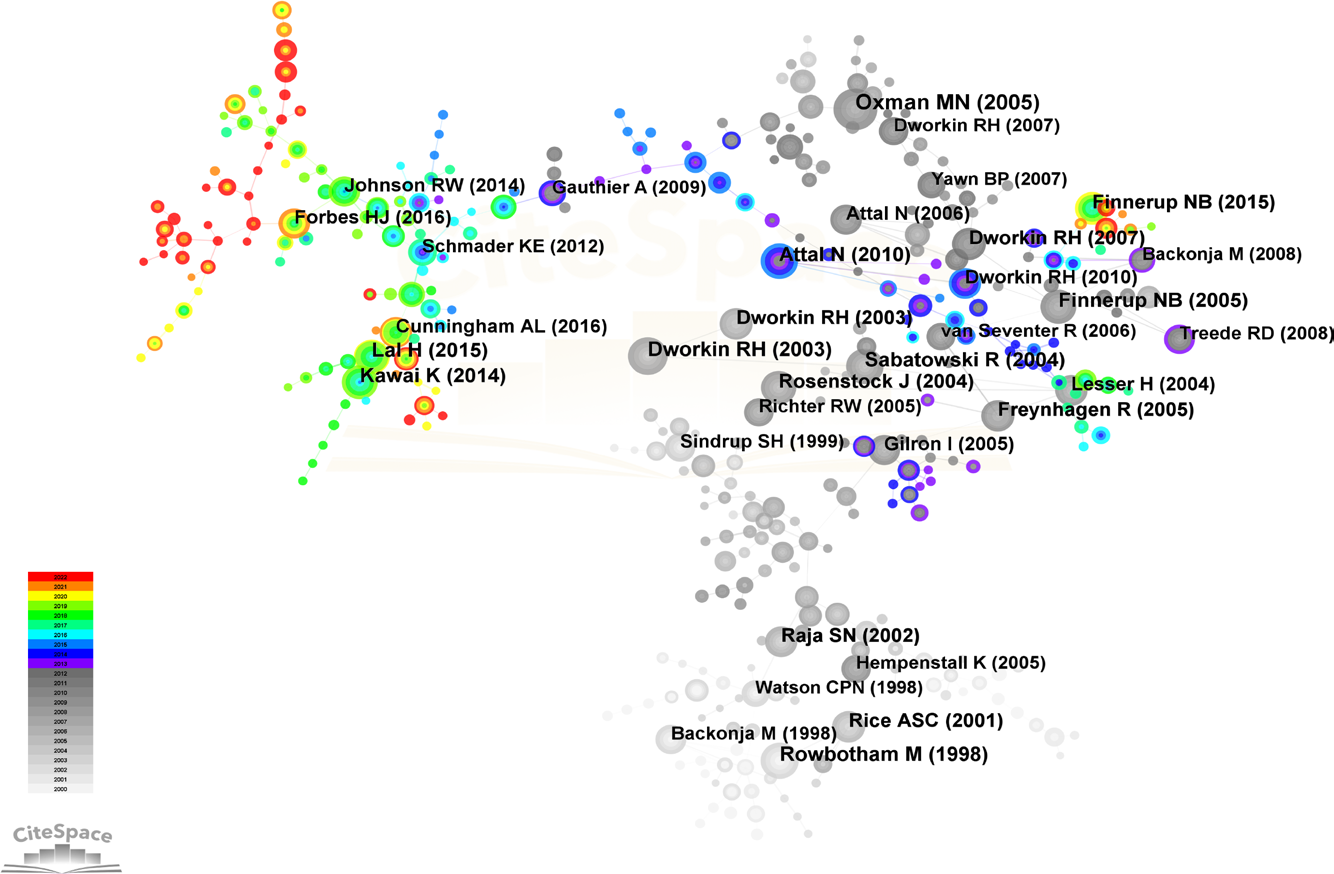
Network map of references from Citespace. Each node represents a citation and the size of the node presents the number of that citation. The thickness of the lines indicates the intensity of the relationship between the regions/countries, which are represented by the connections between the nodes. The gray ring in the inner circle represents publications from the earliest year, while the red ring in the outer circle represents publications from the most recent year.

**Figure 13.**
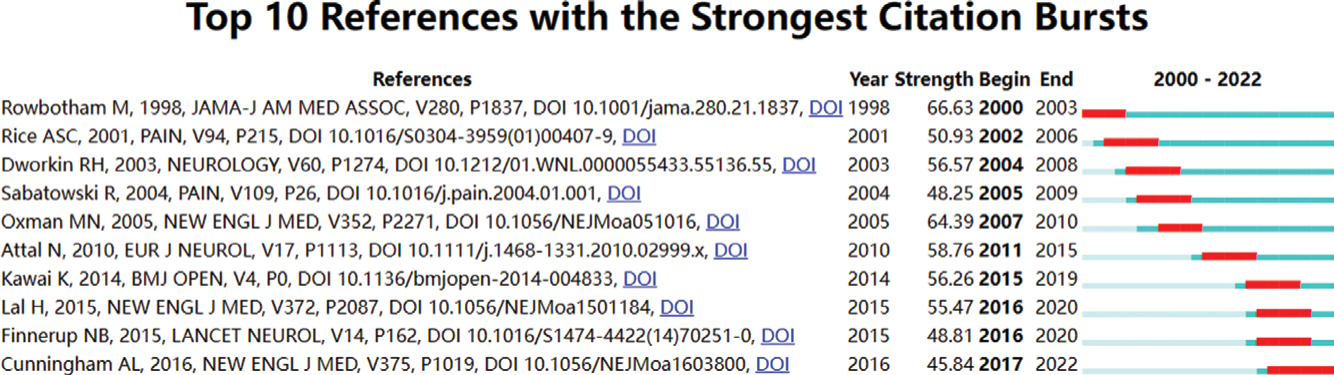
Top 10 references with the strongest citation bursts about PHN. PHN = postherpetic neuralgia.

The implementation of co-citation analysis within references aids in determining the frequently utilized sources and assesses the connections between them. Figure 12 and Table 8 presented the top 10 co-cited references. The article published by Oxman MN in the *New England Journal of Medicine* received the most PHN-related references (165), followed by the article published by Dworkin RH in the *Neurology* (129), the article published by Rowbotham M in the *JAMA-Journal of the American Medical Association* (120), the article published by Sabatowski R in the *Pain* (118), and the article published by Attal N in the *European Journal of Neurology* (116). The references mentioned above had a significant impact on the research areas of PHN. Figure 13 displayed the top ten references that have demonstrated the most prominent citation bursts. The publication wrote by Cunningham, AL in the *New England Journal of Medicine* as depicted in Figure 12, has generated significant attention in contemporary discourse.

**Table 8:** Top 10 citations for publications on PHN.

Rank	Title	Frequency	Journal	JCR	IF (2021)	First Author	Year	Centrality
1	A vaccine to prevent herpes zoster and postherpetic neuralgia in older adults	165	New England Journal of Medicine	Q1	176.079	Oxman MN	2004	0.84
2	Pregabalin for the treatment of postherpetic neuralgia - A randomized, placebo-controlled trial	129	Neurology	Q1	11.800	Dworkin RH	2003	0.01
3	Gabapentin for the treatment of Postherpetic Neuralgia A Randomized Controlled Trial	120	JAMA-Journal of the American Medical Association	Q1	157.335	Rowbotham M	1998	0.03
4	Pregabalin reduces pain and improves sleep and mood disturbances in patients with post-herpetic neuralgia: results of a randomized, placebo-controlled clinical trial	118	Pain	Q1	7.926	Sabatowski R	2004	0.05
5	EFNS guidelines on the pharmacological treatment of neuropathic pain: 2010 revision	116	European Journal of Neurology	Q1	6.288	Attal N	2010	0.03
6	Systematic review of incidence and complications of herpes zoster: towards a global perspective	114	BMJ Open	Q2	3.006	Kawai K	2014	0.06
7	Algorithm for neuropathic pain treatment: An evidence based proposal	111	Pain	Q1	7.926	Finnerup NB	2005	1.03
8	Efficacy of an Adjuvanted Herpes Zoster Subunit Vaccine in Older Adults	110	New England Journal of Medicine	Q1	176.079	Lal H	2015	0.11
9	Gabapentin in postherpetic neuralgia: a randomized, double blind, placebo controlled study	101	Pain	Q1	7.926	Rice ASC	2001	0.02
10	Pregabalin for the treatment of painful diabetic peripheral neuropathy: a double-blind, placebo-controlled trial	99	Pain	Q1	7.926	Rosenstock J	2004	0.03

PHN = postherpetic neuralgia.

## 4. Discussion

PHN is a well-known type of intractable neuropathic pain,^[31]^ which is the most common and severe complication of HZ.^[32]^ The reason for the pain of PHN is that HZ is reactivated by the varicella-zoster virus that is latent in the body. The virus reaches the affected area along the descending sensory nerve, destroying the peripheral nerve tissues such as the dorsal root ganglia and the peripheral nerve, causing local tissue damage and inflammatory reaction, sensitizing peripheral nociceptors, and then developing into central sensitization, causing spontaneous pain and hyperalgesia.^[33]^ The current guidelines believe that neuroplasticity is the basis of PHN generation, and its mechanism may include peripheral and central sensitization and a series of pathophysiological changes such as abnormal increase in the excitability of related neurons or enhanced synaptic transmission that cause the pain threshold to decrease and pain signal amplification.^[34]^ Half of the patients who are more than 50 years have the risk of developing PHN.^[35]^ It is characterized by persistent skin burning or knife-like neuralgia, hyperalgesia, and allodynia in the affected area, which greatly affects quality of life.^[36,37]^ Nowadays, treatment for PHN is based exclusively on symptom control and targeting the mechanisms causing pain.^[38]^ The goal of PHN treatment is to improve quality of life by relieving pain. However, the pain experienced in PHN is often refractory to therapy, with as many as half of patients failing to respond to any treatment; other patients may experience limited efficacy despite being on multiple agents.^[39]^ Currently, the main strategies for PHN management are medication and invasive interventional therapies. Pharmacological agents include opioids, antiviral drugs, nonsteroidal anti-inflammatory drugs, antiepileptic drugs, anticonvulsants, tricyclic antidepressants, and invasive interventional therapies, including pulsed radiofrequency of the dorsal root ganglion, electrical stimulation of the spinal cord, morphine pump implantation, peripheral neurotomy, autologous fat grafting, and acupuncture therapy.^[40–42]^ However, some of these approaches have many adverse effects and risks, such as respiratory depression, nausea, vomiting, addiction, allergy, bleeding, infection, pneumothorax, and spinal cord injury. Therefore, it is important to find another effective and safe treatment for PHN.^[36]^ To sum up, it is crucial to conduct a thorough evaluation of the current research status in this particular field, while also pinpointing areas that exhibit significant levels of activity and emerging research patterns. This study is a novel endeavor that employs visual analysis methods to scrutinize scholarly articles published in the last twenty-three years. The objective is to perform qualitative and quantitative evaluations of publications related to PHN.

### 4.1. General information

The results of our study suggested that there has been a notable increase in academic attention towards PHN, as demonstrated by the steady growth in the number of research publications on the topic worldwide between 2000 and 2022. Based on the data presented in Figure 2, it was observed that there was a consistent increase in the yearly publication output from 2002 to 2010. Subsequently, there has been a gradual stabilization in the annual publication volume. This statement pertains to several trials conducted circa 2002, which concluded that certain medications can effectively mitigate the pain associated with PHN.^[43–45]^ The trend under observation may be linked to the increased prevalence of PHN and the causative factors that contribute to its development. Based on the aforementioned trends, it is suggested that PHN has received substantial scholarly interest and is expected to maintain its importance as a subject of study in the coming years.

According to our research, the USA has emerged as the most productive and academically influential country. In comparison to the USA, other countries exhibited a lower number of publications. The statistical analysis suggested that the USA is the leading contributor to the field of PHN. The government allocation of funds towards healthcare can be considered a crucial metric for evaluating the efficacy of medical research.^[46]^ The USA demonstrates a comparatively elevated level of healthcare expenditure, with an average yearly spending of $10,202 per inhabitant, surpassing that of other countries. This particular characteristic could possibly explain the country higher count of publications in the respective field.^[47]^ Based on our research, Argentina demonstrated the most significant level of collaboration with other countries, trailed by Panama and Philippines. While some countries and regions demonstrated collaborative efforts in the field of PHN research, a significant number of them did not engage in such international collaboration. The findings indicated that it is crucial to promote inter-organizational collaboration across nations in order to facilitate the global advancement of research on PHN. Consequently, it has been determined that there is a need to strengthen international collaboration.

In terms of journals and co-cited Journals, the *Pain* has demonstrated a significant level of focus in the field of PHN, as evidenced by its recent publication of a substantial number of PHN-related papers. This journal serves as a valuable platform for academic discourse and exchange. The *Pain* is an international medical journal that covers a wide range of clinical, basic, and translational research in the field of pain, including PHN. An examination of the characteristics of highly productive journals can facilitate comprehension of present-day patterns.^[48]^ Our study revealed that a considerable proportion of research on PHN exhibited a multidisciplinary nature and had been disseminated through reputable scholarly outlets. The preponderance of the cited references emanated from reputable scholarly journals, with the *Pain* being also the most frequently cited publication. The aforementioned proposition implied that scholars ought to broaden their avenues for disseminating their PHN research outcomes in top-tier academic publications in order to enhance the global prominence of PHN.

Regarding authors and co-cited authors, it can be observed that Baron, Ralf from Univ Klinikum Schleswig Holstein in the Germany has exhibited the highest level of productivity and influence. This is indicative of the extensive efforts that he and his team have dedicated to the field of PHN over the past 23 years. His primary area of focus was centered on the utilization of Lidocaine for the purpose of pain management in patients with PHN, as well as the significance of sensory symptom characteristics about PHN.^[49,50]^ The individual with the highest number of citations was DWORKIN RH. His investigation encompasses various facets, including the diagnosis, prevention, treatment, and risk factors associated with PHN.^[51]^ The data presented in Table 5 and Figure 6 indicated that the vast majority of authors collaborated with colleagues who were based in their country of origin. The level of international collaboration among authors in this instance was suboptimal. The elimination of language barriers among authors from diverse nations is imperative for enhancing global cooperation in this field.

### 4.2. Research hotspots and trends

Prominent research areas within this specific field entail the analysis of burst in keyword usage and the patterns of keyword co-occurrence distribution. The application of keyword or reference co-occurrence analysis, clusters, and bursts in citations over time has been regarded as effective methodologies for detecting research hotspots or emerging trends.^[24]^ According to the findings presented in Table 7 and Figure 11, the 5 most frequently employed keywords were “postherpetic neuralgia,” “neuropathic pain,” “herpes zoster,” “double blind,” and “efficacy.” Given that PHN falls under the category of neuropathic pain,^[52]^ the terminology employed by the authors predominantly aligns with the keywords featured in the article title. The preponderance of the aforementioned keywords could potentially be attributed to this phenomenon. The most recent burst of keywords included “pulsed radiofrequency,” “prevention,” “herpes zoster,” and “dorsal root ganglion.” It is suggested that pulsed radiofrequency is a novel and secure approach to pain management.^[53]^ Specifically, it has been increasingly utilized for the relief of PHN in recent years, demonstrating both safety and efficacy.^[54]^ For “prevention,” the prevention of PHN has been a prominent area of research in recent years, with a focus on interventions such as vaccination,^[55]^ gabapentinoids,^[56]^ epidural block.^[57]^ For “herpes zoster,” the occurrence of PHN is causally associated with HZ. For “dorsal root ganglion,” the spinal cord stimulation has been employed for more than half a century in the management of pain syndrome associated with the dorsal root ganglion. Numerous studies have demonstrated the efficacy of spinal cord stimulation as an analgesic. In recent times, there has been a surge in the utilization of dorsal root ganglion stimulation as a prevalent approach to managing PHN. This method has been established as a viable treatment alternative for patients who exhibit resistance to pharmacological interventions for PHN.^[26]^ Figure 11 revealed that the terms “safety” and “risk” exhibited a prolonged duration, suggesting that the identification of treatment alternatives that offer elevated safety levels and mitigate risk factors for PHN has consistently been a subject of interest. Table 7, Figure 11, and Figure 12 indicated that pharmacological treatment, clinical trials, diagnosis, and pathogenesis have not been the focus of recent research.

Utilizing current research developments, we will now analyze the fundamental elements that give rise to these central domains and trends, as outlined below: Firstly, the primary aim of treatment should be to avert the onset of PHN. This can be achieved through the use of antiviral drugs,^[58]^ temporary spinal cord stimulation,^[59]^ and vaccination,^[55]^ which have demonstrated efficacy in this regard. Secondly, the investigation of the risk factors associated with the onset of PHN holds significant importance in terms of preventive measures. Recent studies have identified several independent risk factors for PHN, including age greater than or equal to 50, lesions on the upper limbs and shoulders, hypertension, asthma, diabetes, smoking, underweight or obesity, female gender, and immunosuppressive treatments.^[13,60]^ Thirdly, the pursuit of efficacious pain management remedies has consistently been a focal point in this field. Currently, the efficacy of traditional analgesics, such as topical lidocaine patches or low-dose capsaicin for external application, in treating PHN remains inconclusive. In certain countries, the utilization of tricyclic antidepressants, including desipramine, is prohibited in this context due to their associated unfavorable effects, which encompass dryness of the oral cavity, constipation, weight gain, visual impairment, and orthostatic hypotension.^[61]^ The utilization of pulsed radiofrequency and dorsal root ganglion stimulation as novel pain management techniques has yielded favorable results in terms of achieving optimal analgesia and enhancing quality of life. As such, further comprehensive investigation is warranted.^[58]^

Figures 10 and 11 showed that “#0 herpes zoster” was the largest cluster term which encompassed studies on pain and analgesics. “#1 diabetic neuropathy” was the second most common cluster term. Individuals diagnosed with diabetes are susceptible to contracting zoster infection and PHN as a result of their weakened immune system.^[62]^ Regarding “#2 quality of life”, it is noteworthy that PHN can significantly impact the daily activities and functioning of individuals. As such, enhancing the quality of life of patients afflicted with PHN is of paramount significance. #2 comprised a variety of techniques for managing pain.

The characteristics under consideration have been identified through the utilization of references in the current study. The significance of pain management for PHN is noteworthy within the scope of this research field, as evidenced by its elevated ranking (1–5, 8–10) in Table 8. Considerable focus has been directed towards examining alterations in the frequency of HZ and its associated complications, as indicated by its placement as the 6th highest ranked item in Table 8. The assessment standards pertaining to the analgesic effectiveness of PHN hold significant importance, as indicated by their ranking of 7 in Table 8. As previously stated, advanced age constitutes a significant risk factor for the development of PHN. Therefore, effective pain management strategies for PHN must prioritize the elderly population, as evidenced by their ranking of 1 and 8 in Table 8. Pharmacological and immunological interventions continue to be the favored modalities for managing PHN, as indicated by their high ranking (1, 2, 3, 4, 8, 9, and 10) in Table 8. Furthermore, it is imperative to prioritize the enhancement of the patient quality of life when treating PHN, as indicated by its ranking of 4 in Table 8. The article authored by Cunningham AL and published in the *New England Journal of Medicine* has garnered significant attention in recent times. The study primarily investigated the effectiveness of the HZ subunit vaccine in adults aged 70 years or above, as depicted in Figure 13.

### 4.3. Strengths and limitations

The present investigation provided scholarly guidance on the process of selecting hotspots and identifying potential collaborators who share relevant research interests. Scholars can effectively select scholarly articles that are pertinent to their research domains by utilizing the abstracts of significant references and periodicals that have been furnished.

There exist several constraints that hinder the progress of this study. The primary utilization of WOS Core Collection as the leading database for bibliometric analysis restricted the examination of other databases, potentially leading to the omission of pertinent research material. Secondly, in order to mitigate the potential for bias, our analysis excluded articles authored in languages other than English and solely incorporated periodicals composed in English. Thirdly, it is plausible that the scope of our study may have omitted recent papers with significant impact but low citation frequency. Notwithstanding the aforementioned constraints, our investigation provides a comprehensive survey of the existing literature on PHN, which has the capacity to illuminate the current state and prospective trajectory of the field.

## 5. Conclusion

The present investigation utilized a bibliometric approach to conduct a thorough examination of scholarly works related to PHN. The analysis encompassed diverse facets, including the quantity of published literature, geographic dispersion, academic periodicals, authorship, keywords, and references. These findings suggested that PHN has garnered the attention of academics globally, highlighting the need for increased collaboration among scholars from different countries. The present investigation has revealed that the existing literature on PHN is primarily focused on identifying the risk factors associated with the condition, devising preventive measures, and exploring novel and secure pain management techniques. Notably, the research has highlighted the efficacy of pulsed radiofrequency, dorsal root ganglion stimulation, and vaccination in this regard. In recent years, there has been a decrease in interest in pharmacological treatment, clinical trials, diagnosis, and pathogenesis. Henceforth, future research endeavors in the realm of PHN should focus on risk factors, prevention, and the utilization of novel pain management techniques, as opposed to the diagnosis, pathogenesis, and pharmacotherapy of PHN. To conclude, our study offers insights that may be valuable to scholars in this area, enhancing their comprehension of current areas of focus and advancements in the field of public health nursing.

## Acknowledgments

The author would like to thank Professor Furui Miao and Yushan Fan for their help in this study.

## Author contributions

**Conceptualization:** Yujun He.

**Data curation:** Yujun He, Jiujie He, Fangzhi Zhang, Pu Yang.

**Funding acquisition:** Yushan Fan.

**Formal analysis:** Yujun He, Jiujie He, Fangzhi Zhang, Zibin Wang, Pu Yang.

**Investigation:** Yujun He, Jiujie He, Furui Miao, Zibin Wang, Yiping Zhao.

**Methodology:** Yushan Fan, Yiping Zhao, Pu Yang.

**Project administration:** Furui Miao, Yushan Fan, Fangzhi Zhang.

**Resources:** Jiujie He, Furui Miao, Fangzhi Zhang, Zibin Wang, Yu Wu.

**Software:** Jiujie He, Yiping Zhao.

**Supervision:** Furui Miao, Yushan Fan, Yu Wu.

**Validation:** Yujun He.

**Visualization:** Yujun He, Fangzhi Zhang, Zibin Wang, Yu Wu, Yiping Zhao.

**Writing – original draft:** Yujun He.

**Writing – review & editing:** Furui Miao, Yushan Fan.
